# 

**Published:** 1905-02-25

**Authors:** 


**NOTICE TO CORRESPONDENTS.**

All MSS., Letters, Books for Review, and other matters intended for the Editor should be addressed The Editor, "The Hospital," The Hospital Buildings, 28 & 29 Southampton Street, Strand, W.C.

The Editor cannot undertake to return rejected MS., even when accompanied by stamped directed envelope.

**Notice to Advertisers and Subscribers.**

All Advertisements, Orders for Copies of The Hospital, and Business Communications should be addressed to **THE MANAGER (Not to the Editor)**, The Hospital, 28 & 29 Southampton Street, Strand, London, W.C.

The Cost of Subscription, post free, is as follows:—Per week, 3½d.; per quarter, 3s. 3d.; per half-year, 6s. 6d.; per annum, 13s. Foreign and Colonial:—Per annum, 19s. 6d., payable, in all cases, in advance.

**NOTICE.—Vol. XXXVI. of "The Hospital" (April to September 1904), in handsome cloth binding, is now on sale at the office of this Journal, price 10s. 6d. Cases for binding the Numbers contained in this Volume 2s. Index to Vol. XXXVI., 3½d., post free.**

**The Monthly part for February is now ready. Price 1s., by post, 1s. 3d.**

### BOOKS RECEIVED.

Baillière, Tindall and Cox.

"Lateral Curvature of the Spine." Sixth edition. By R. Barwell, F.R.C.S.

"Pulmonary Consumption." Second edition. By A. Latham, M.D.. F.R.C.P.

The Scientific Press, Ltd.

"Burdett's Hospitals and Charities, 1905."

"Elements of Anatomy and Physiology." By W. Bernard Secretan, F.R.C.S.

University Tutorial Press.

"Matriculation Directory." No. XXXIX. January 1905.

J. B. Lippincott Co.

"International Clinics." Vol. IV. Fourteenth Series.

Cassell and Co., Limited.

"The Open-air Treatment of Pulmonary Consumption." By F. W. Burton-Fanning, M.D.

The Practitioner, Limited.

"Congenital Dislocation of the Hip." By J. Jackson Clarke, M.B., F.R.C.S.

A. C. Fifield.

"Edward Carpenter, Poet and Prophet." By Ernest Crosby.

J. Wright & Co.

"Diseases of the Stomach and Intestines." By Boardman Reed, M.D.

"Naked Eye Anatomy of the Human Teeth." By T. E. Constant, M.R.C.S, L.R.C.P.

J. Murray.

"The Teachings of Zoroaster." By S. A. Kapadia.

### MEDICAL APPOINTMENTS.

**John F. H. Broadbent**, M.D.Oxon., Physician in Charge of Out-patients at St. Mary's Hospital, Paddington, W. **A. Brown**, L.R.C.P.I., M.R.C.S.Eng., Certifying Surgeon under the Factory and Workshop Act for the Masham District of the county of York. **Ada M. Browne**, L.S.A., Anæsthetist to the Chelsea Hospital for Women. **F. F. Burghard**, M.D., M.S.Lond., F.R.C.S Eng., Consulting Surgeon to the Bromley Cottage Hospital. **F. H. Burton-Brown**, M.D.Oxon., Clinical Assistant to the Chelsea Hospital for Women. **P. F. Doorly**, L.R.C.P., L.R.C.S.Edin., L.F.P.S.G., Certifying Surgeon under the Factory and Workshop Act for the Carrick-on-Shannon No. 2 District of the county of Roscommon. **J. M. Fortescue-Brickdale**, M.A., M.D.Oxon., Out-patient Physician and Pathologist to the Royal Hospital for Sick Children and Women, Bristol. **Trevethan Frampton**, F.R.C.P.Edin., Clinical Assistant to the Chelsea Hospital for Women. **Arthur Sydney Gedge**, M.R.C.S., L.R.C.P.Lond., Medical Officer for the Fifth District of the Devizes Union. **Wilfred J. Harris**, M.D.Cantab., Physician in Charge of Out-patients at St. Mary's Hospital, Paddington, W. **P. Maynard Heath**, B.S, F.R.C.S., Surgical Registrar to University College Hospital. **A. M. Laurie**, L.R.C.P. & S Edin., Parochial Medical Officer and Public Vaccinator for the joint parishes of Houston and Killellan, Renfrewshire. **W. J. Lawrie**, M.D., C.M.Glasg., Certifying Surgeon under the Factory and Workshop Act for the Ayr District of the county of Ayr. **H. Willoughby Lyle**, M.D., B.S.Lond., F.R.C.S.Eng., Consulting Ophthalmic Surgeon to the Bromley Cottage Hospital. **A. P. Percival**, M.B., C.M.Edin., Certifying Surgeon under the Factory and Workshop Act for the Knottingley District of the county of York. **Walter Sykes**, L.R.C.P., L.R.C.S., L.F.P.S.G., Honorary Ophthalmic and Aural Surgeon to the Preston and County of Lancaster Royal Infirmary.

ST. THOMAS'S HOSPITAL.—The following House appointments have been made to date from Tuesday, March 7th, 1905:—Resident House Physicians: **R. E. Whitting**, M.A., M.B., B.C.Cantab.; **F. A. Brodribb**, M.R.C.S., L.R.C.P.; **A. G. Gibson**, B.A., M.B., B.Ch.Oxon., B.Sc.Lond. (*Extension*); **K. Takaki**, M.R.C.S., L.R.C.P. (*Extension*). House Physicians to Out-patients: **H. R. Dean**, B.A., M.B., B.Ch.Oxon., M.R.C.S., L.R.C.P.; **A. C. Inman**, M.A., M.B., B.Ch.Oxon. Resident House Surgeons: **H. A. Kisch**, M.R.C.S., L.R.C.P.; **G. R. Footner**, B.A.Cantab., M.R.C.S., L.R.C.P.; **R. E. G. Gray**, M.A.Cantab., M.R.C.S., L.R.C.P.; **J. C. F. D. Vaughan**, M.R.C.S., L.R.C.P. House Surgeons to Out-patients: **L. E. C. Norbury**, M.R.C.S., L.R.C.P.; **D. K. Coutts**, M.R.C.S., L.R.C.P.; **J. H. Bletsoe**, M.B., B.S.Lond., M.R.C.S., L.R.C.P.; **R. J. C. Thompson**, M.R.C.S., L.R.C.P. Obstetric House Physicians: **E. W. Parry**, M.R.C.S., L.R.C.P. (*Senior*); **N. C. Carver**, B.A., B.C.Cantab., M.R.C.S., L.R.C.P. (*Junior*). Ophthalmic House Surgeons: **A. B. Bradford**, M.B., B.S.Durh., M.R.C.S., L.R.C.P. (*Senior*); **F. R. E. Wright**, M.B.Lond., M.R.C.S., L.R.C.P., D.P.H. (*Junior*). Special Departments: (Throat) **W. G. Howarth**, B.A.Cantab., M.R.C.S., L.R.C.P.; **G. J. Langley**, M.B., B.S.Lond.; (Skin) **W. Haward**, M.B., B.S.Durh., M.R.C.S., L.R.C.P.; **N. Carpmael**, F.R.C.S., L.R.C.P.; (Ear) **J. H. Drew**, M.R.C.S., L.R.C.P.; **C. Ll. Morgan**, M.R.C.S., L.R.C.P. Electrical Department: **F. Fowler**, M.R.C.S., L.R.C.P., L.S.A. X-ray Department: **H. M. Thomas**, M.R.C.S., L.R.C.P.

**"The Hospital" Institutional Library.**

"Hospitals and Asylums of the World." 4 Vols., with a Portfolio of Plans, complete £12 12s.
"Burdett's Hospitals and Charities, 1904." 5s. net.
"Burdett's Official Nursing Directory." 8s. net.
"Cottage Hospitals, General, Fever, and Convalescent." 10s. 6d.
"Uniform System of Accounts for Hospitals and Public Institutions." New Edition. 4s. net.
"Hospital Expenditure: The Commissariat." 2s. 6d.
"A Legal Handbook for the Use of Hospital Authorities." 2s. 6d.
"The Cottage Hospital Case Book or Register of Patients." 10s. and 12s. 6d.

All these are published by the SCIENTIFIC PRESS, Ltd., and may be obtained through any bookseller, or direct from the publishers, 28 and 29 Southampton Street, Strand, London, W.C.

### Medical Appointments.

**PRACTICAL GUIDE TO SURGICAL BANDAGING AND DRESSINGS.**
By Wm. Johnson Smith, F.R.C.S.
Demy 16mo, profusely illustrated (suitable for apron pocket), cloth - - - 2/-
Post free

**ELEMENTARY ANATOMY AND SURGERY FOR NURSES.**
By William McAdam Eccles, M.S. Lond., M.B., F.R.C.S., L.R.C.P.
Crown 8vo, profusely illustrated, cloth 2/6
Post free

**ELEMENTARY PHYSIOLOGY FOR NURSES.**
By C. F. Marshall, M.D., B.Sc., F.R.C.S.
Crown 8vo, illustrated, cloth • 2/-
Post free

**OPHTHALMIC NURSING.**
By Sydney Stephenson, M.B., F.R.C.S.E.
New and Revised Edition. Crown 8vo, profusely illustrated with Original Drawings, list of Instruments required, and a Glossary of Terms, cloth - 3/6 net
3/10
Post free

**NURSING IN DISEASES OF THE THROAT, NOSE AND EAR.**
By P. Macleod Yearsley, F.R.C.S. Eng., M.R.C.S., L.R.C.P. Lond.
Demy 8vo, illustrated, cloth • • • 2/6
Post free

**ART OF MASSAGE.**
By A. Creighton Hale.
Second Edition, Demy 8vo, 150 pp., profusely illustrated with over 70 special Cuts, cloth gilt - - - - - 6/-
Post free

**A COMPLETE HANDBOOK OF MIDWIFERY.**
By J. K. Watson, M.D. Edin., Author of "A Handbook for Nurses."
Crown 8vo, over 350 pp., profusely illustrated with 143 diagrams - - - 6/- net
6/4
Post free

**THE MODERN NURSING OF CONSUMPTION.**
By Dr. Jane H. Walker.
Crown 8vo, limp cloth • • • - 1/- net
1/2
Post free

**SURGICAL WARD-WORK AND NURSING.**
By Alexander Miles, M.D. Edin., C.M., F.R.C.S.E.
Second Edition, Enlarged and entirely Revised. Demy 8vo, copiously illustrated, cloth gilt - - - - - 3/6 net
3/10
Post free

**MODERN NURSING IN PRIVATE PRACTICE.**
By Sir William Bennett, K.C.V.O., F.R.C.S.
Paper covers - - - - - 6d. net
7d.
Post ree

**ON PREPARATION FOR OPERATION IN PRIVATE HOUSES.**
By Stanmore Bishop, F.R.C.S.
Crown 8vo, paper covers - - - 6d.
Post free

**THE EXAMINATION OF THE URINE.**
By J. K. Watson, M.D., M.B., C.M., Author of "A Handbook for Nurses."
Demy 16mo, cloth boards - - 1/- net
1/-
Post free

**COMPLETE CATALOGUE OF NURSING MANUALS POST FREE ON APPLICATION.**

**The Scientific Press, Ltd.,**
**Publishers of Nursing Text-Books, Case-Books and Charts of all descriptions.**
**28 & 29 SOUTHAMPTON STREET, STRAND, LONDON, W.C.**

